# Evaluation of *Strongyloides stercoralis* infection in patients with HTLV-1

**DOI:** 10.7705/biomedica.5888

**Published:** 2022-03-01

**Authors:** Nilo Manoel Pereira Vieira Barreto, Marina Morena Brito Farias, Cíntia de Lima Oliveira, Weslei Almeida Costa Araujo, Maria Fernanda Rios Grassi, Joelma Nascimento de Souza, Beatriz Soares Jacobina, Márcia Cristina Aquino Teixeira, Bernardo Galvão-Castro, Neci Matos Soares

**Affiliations:** 1 Instituto de Ciências da Saúde, Programa de Pós-graduação em Processos Interativos dos Órgãos e Sistemas, Universidade Federal da Bahia, Bahia, Brasil Universidade Federal da Bahia Instituto de Ciências da Saúde Programa de Pós-graduação em Processos Interativos dos Órgãos e Sistemas Universidade Federal da Bahia Bahia Brazil; 2 Departamento de Análises Clínicas e Toxicológicas, Faculdade de Farmácia, Universidade Federal da Bahia, Bahia, Brasil Universidade Federal da Bahia Departamento de Análises Clínicas e Toxicológicas Faculdade de Farmácia Universidade Federal da Bahia Bahia Brazil; 3 Instituto Gonçalo Moniz, Fundação Oswaldo Cruz, Bahia, Brasil Instituto Gonçalo Moniz Fundação Oswaldo Cruz Bahia Brasil; 4 Centro de HTLV, Escola Bahiana de Medicina e Saúde Pública-BAHIANA, Bahia, Brasil Centro de HTLV Escola Bahiana de Medicina e Saúde Pública-BAHIANA Bahia Brasil

**Keywords:** Strongyloides stercoralis, strongyloidiasis, human T-lymphotropic virus 1, coinfection, helminths, Strongyloides stercoralis, estrongiloidiasis, virus linfotrópico T de tipo 1 humano, coinfección, helmintos

## Abstract

**Introduction:**

Individuals infected with the human T-lymphotropic virus type 1 (HTLV-1) may present severe and disseminated forms of *Strongyloides stercoralis* infection with low therapeutic response.

**Objective:**

To investigate the *S. stercoralis* infection and the seroprevalence of IgG anti-*S. stercoralis* antibodies in individuals infected with HTLV-1 attending the Reference Center for HTLV-1 (CHTLV) in Salvador, Bahia, Brazil.

**Materials and methods:**

We conducted a cross-sectional study in 178 HTLV-1-infected individuals treated at the HTLV specialized center between January, 2014, and December, 2018. The parasitological diagnosis of *S. stercoralis* was performed using the Hoffman, Pons and Janer, agar plate culture, and Baermann-Morais methods. The IgG anti-*S. stercoralis* detection was performed using an *in house* enzyme-linked immunosorbent assay (ELISA). The HTLV-1 infection was diagnosed using a commercial ELISA and confirmed by Western blot.

**Results:**

The frequency of *S. stercoralis* infection was 3.4% (6/178). Individuals infected with *S. stercoralis* from rural areas (50.0%; 3/6) also showed *S. stercoralis* hyperinfection (>3,000 larvae/ gram of feces). The frequency of circulating anti-*S. stercoralis* IgG antibodies was 20.8% (37/178).

**Conclusions:**

HTLV-1-infected people living in precarious sanitary conditions are more prone to develop severe forms of *S. stercoralis* infection. Considering the high susceptibility and unfavorable outcome of the infection in these individuals, the serological diagnosis for *S. stercoralis* should be considered when providing treatment.

## Resumen

**Introducción.:**

Los individuos infectados por el virus linfotrópico T humano tipo 1 (HTLV-1) pueden presentar formas graves y diseminadas de infestación por *Strongyloides stercoralis* con poca mejoría terapéutica.

**Objetivo.:**

Investigar la infestación por *S. stercoralis* y la seroprevalencia de IgG anti-*S. stercoralis* en individuos infectados por HTLV-1 atendidos en el Centro de Referencia para HTLV-1 (CHTLV), en Salvador, Bahía, Brasil.

**Materiales y métodos.:**

Se hizo un estudio transversal con 178 individuos infectados por HTLV-1 atendidos en el centro especializado de HTLV entre enero de 2014 y diciembre de 2018. El diagnóstico parasitológico de *S. stercoralis* se hizo mediante los métodos de Hoffman, Pons y Janer, cultivo en placa de agar y Baermann-Morais. Para la detección de IgG anti-*S. stercoralis*, se utilizó una prueba casera de inmunoabsorción ligada a enzimas (ELISA). La infección por HTLV-1 se diagnosticó usando un ELISA comercial y se confirmó mediante *Western blot*.

**Resultados.:**

La frecuencia de infestación por *S. stercoralis* fue del 3,4 % (6/178). Además, los individuos infestados por *S. stercoralis* provenientes de la zona rural (50,0 %; 3/6) también mostraron hiperinfestación por *S. stercoralis* (>3.000 larvas/gramo de heces). La frecuencia de anticuerpos IgG anti-*S. stercoralis* fue del 20,8 % (37/178).

**Conclusiones.:**

Las personas infectadas por HTLV-1 que viven en condiciones sanitarias precarias son más propensas a desarrollar formas graves de infestación por *S. stercoralis.* Teniendo en cuenta la gran vulnerabilidad y el resultado desfavorable de la infección en estos individuos, se debe considerar el diagnóstico serológico de *S. stercoralis* para administrar el tratamiento.

Strongyloidiasis, a neglected tropical disease that affects around 370 million people worldwide, is caused by soil-transmitted helminths of the genus *Strongyloides* mostly distributed throughout tropical and subtropical regions ^1^^-^^3^. *Strongyloides stercoralis*, the most common agent of this disease, is classified according to its prevalence: sporadic (<1%), endemic (1-5%), or hyperendemic (> 5%) (Pires, Dreyer, 1993). Hyperendemic areas are located mainly in the tropics, especially in the developing countries of Asia, Sub-Saharan Africa, and Latin America (notably Brazil and Colombia) ^4^^,^^5^. In Brazil, the average rate of *S. stercoralis* infection between 1990 and 2009 was approximately 5.5%, which means the country is hyperendemic ^6^. In Salvador, the capital city of the state of Bahia, Brazilian Northeast*,* the prevalence of infection ranges from 4.6% to 6.6% ^7^^,^^8^.

In the general population, *S. stercoralis* infection can be characterized as chronic or asymptomatic. However, immunosuppressed individuals, such as those infected with human T-lymphotropic virus type 1 (HTLV-1), have a greater susceptibility to infection, which can progress to life-threatening forms of strongyloidiasis ^9^^-^^11^ and poor therapeutic response ^12^^,^^13^.

Five to ten million people are infected with HTLV-1 worldwide ^14^. Brazil is the country with the highest absolute number of HTLV-1 cases, about 800,000 ^15^. In a study conducted in Salvador, the overall prevalence of HTLV-1 infection was 1.74%, which increases significantly in females over 51 years of age reaching up to 9% ^16^^,^^17^. Previous studies have reported elevated frequencies of *S. stercoralis* in patients infected with HTLV-1 from Japan ^12^^,^^18^. In Brazil, with the highest number of HTLV-1 carriers, the frequency of *S. stercoralis* infection varies according to the geographic region with greater occurrence in the North and Northeast regions ^19^ where it ranges from 12 to 15.7% ^9^^,^^20^^,^^21^.

The HTLV-1 transmission may occur in three ways: a) through sexual contact with a 60% efficiency when transmitted from man to woman and 4% from female to male; b) via the blood when sharing syringes or contaminated needles, or by blood transfusion, and c) vertically from mother to child, especially through breastfeeding ^17^^,^^22^. HTLV-1 transmission via organ transplant has also been described and is associated with the development of myelopathy/tropical spastic paraparesis (HAM/TSP) possibly due to the immunosuppression to which these individuals are subjected ^23^^,^^24^.

The present study aimed to investigate the prevalence of *S. stercoralis* infection and the seroprevalence of IgG anti-*S. stercoralis* antibodies in individuals infected with HTLV-1 attending the Reference Center for HTLV-1 (CHTLV) in Salvador, Bahia, Brazil.

## Materials and methods

### Study description

The present cross-sectional study was conducted in 178 HTLV-1-infected individuals seen and treated at the Integrated Multidisciplinary Center for HTLV (CHTLV) of the Bahiana School of Medicine and Public Health (EBMSP), in Salvador, Bahia, from January 2014 to December 2018.

CHTLV is a public outpatient clinical center that provides interdisciplinary care and services including general medical treatment, laboratory diagnosis, psychological counseling, and physical therapy. All individuals with associated comorbidities, such as immunosuppression due to the chronic use of glucocorticosteroids, HIV infection, or chronic alcohol abuse, were excluded from the study. The parasitological and immunological diagnoses of *S. stercoralis* were performed at the Faculty of Pharmacy of the Federal University of Bahia, Salvador, Brazil.

### Data and sample collection

A questionnaire was drawn up to collect socio-demographic data and information on individuals’ residential sanitary conditions. Fresh stool samples were obtained from all enrolled subjects and submitted to parasitological examination as described below. Blood samples were collected in tubes containing polymer gel for serum separation and then centrifuged for 10 minutes at 1,620*g*. Sera were frozen at −20°C until use.

### HTLV-1 diagnosis

We screened serum samples for HTLV-1 antibodies at the CHTLV by microparticle CLIA chemiluminescence (Architect rHTLV-1/2, Abbott Diagnostics Division, Wiesbaden, Germany) and confirmed by Western blot following the manufacturer’s instructions (HTLV Blot 2.4, Genelabs Diagnostics, Singapore).

### Strongyloides stercoralis and other intestinal parasites diagnosis

Fresh stool samples from each subject were examined by three different parasitological methods: Hoffman, Pons and Janer ^25^, Baermann-Moraes modified by Rugai ^26^^,^^27^, and agar plate culture (APC) ^28^. The detection of anti-*S. stercoralis* IgG was performed by ELISA ^29^ as described below.

### Larvae quantification

The parasite load was quantified by counting the number of larvae under microscopy (10 x objective lens) found in approximately 1 g of feces using the Baermann-Moraes method. The number of larvae was categorized as “non-quantified” when the parasite was not detected using the Baermann-Moraes method, 1-10, 11-50, 51-100, 101-500, and higher than 500 larvae/g of feces.

### Strongyloides stercoralis antigens for ELISA

*Strongyloides stercoralis* third-stage infective larvae (L_3_) were obtained from the stool of hyperinfested patients. The larvae were cultured in animal charcoal at 28° C for five days and recovered using Rugai’s method ^27^ and then washed 5 times in 0.15 mol/L of phosphate buffered saline (PBS), pH 7.2. Next, parasites were suspended for 5 min in 0.25% sodium hypochlorite and rewashed 5 times in PBS. Larvae were then re-suspended in PBS with protease inhibitors (5 µmol/L EDTA, 1 µmol/L phenyl-methyl sulfonyl fluoride [Sigma], 0.05 µmol/L TPCK/TLCK, 1 µg/ml leupeptin) and sonicated in an ice bath for nine cycles lasting 80s each at 40 kHz (Branson Sonifier Cell Disruptor™, Branson Instruments, Danbury, CT, USA). The larvae homogenate was then centrifuged at 11,000*g* for 30 min at 4 °C, after which the supernatant was collected and analyzed for protein content by the Lowry, *et al*. ^30^ method, divided into aliquots, and stored at −70 °C until use.

### Strongyloides stercoralis IgG-ELISA

The wells of microtiter plates (Corning Inc. Costar polystyrene EIA/RIA plates) were coated with 100 μL of 10 μg/mL *S. stercoralis* antigen in 0.06 mol/L carbonate-bicarbonate buffer, pH 9.6, then incubated overnight at 4 °C and washed 3 times in PBS containing 0.05% Tween-20 (PBS-T). All plates were then blocked with 100 μL PBS-T containing 8% w/v skim milk (PBS-T-Milk) for 1 hour at 37 °C. After the blocking, the wells were washed as described previously. Serum samples diluted at 1:100 in PBS-T-Milk were incubated at 37°C in duplicate in a volume of 100 μL per well for 1 hour. After washing, 100 μL of 1:4000 anti-human IgG conjugated to horseradish peroxidase (Sigma-Aldrich, St. Louis, MO, USA) was added to each plate and incubated under identical conditions. Reactions were visualized by adding substrate, 100 μL of 0.051 mol/L citrate-phosphate buffer (pH 5.0) containing 0.0037 mol/L p-phenylenediamine, and 0.04% hydrogen peroxide followed by a 20 minute incubation period in the absence of light after which 20 μL of 8N sulfuric acid were added to stop the reaction. Absorbance was measured at 450-630 nm on a microplate reader (Awareness Technology, USA).

### Statistical analysis

As the sampling plan was not probabilistic, inferential statistics (hypothesis test and confidence interval) were not used due to the skewed estimate of the standard error ^31^^,^^32^. Data were analyzed using the statistical program IBM SPSS (19.0 for Windows) with quantitative variables being presented in measures of central tendency and dispersion and categorical variables in absolute and relative frequencies.

The cut-off, sensitivity, and specificity for the IgG- anti-*S.stercoralis* ELISA were determined by the receiver operating characteristic curve (ROC) using a total of 81 HTLV-negative sera from 34 samples of individuals infected with *S. stercoralis* (positive controls), 24 without parasitic infections, and 23 who had intestinal parasites other than *S. stercoralis* (negative controls).

### Ethical aspects

This project was approved by the Research Ethics Committee, Faculty of Pharmacy, Federal University of Bahia, under number 2616338. All individuals who agreed to participate in the study signed the Informed Consent Form. Patients diagnosed with *S. stercoralis* and other parasites received prompt treatment.

## Results

### Demographic and socioeconomic characteristics

The mean age of HTLV-1 individuals was 45.60±17.26 years. The majority (65.7%; 117/178) were females from Salvador and the outlying metropolitan area (69.7%; 124/178). Most people (113/178; 63.5%) had a low level of formal education varying from no formal education to incomplete high school and came from low-income families (55.6%; 99/178) receiving between half and a full monthly minimum wage. Regarding their residential sanitary conditions and hygiene habits, most had access to piped water (83.7%; 149/178), sewage system and/or septic tank (88.2%; 157/178) and lived in areas with paved streets (78.1%; 139/178); 16.3% (29/178) of individuals had a habit of walking barefoot (table 1).

**Table 1:** Demographic and socioeconomic characteristics of individuals infected with HTLV-1 (n=178) seen at the Integrated Multidisciplinary Center for HTLV, Salvador, Bahia, Brazil

Variable	Strongyloides stercoralis		Total
	Positive n (%)	Negative n (%)	n (%)
Sex				
	Male	5 (83.3)	56 (32.6)	61 (34.4)
	Female	1 (16.7)	116 (67.4)	117 (65.7)
Age (years)				
	< 20	2 (33.3)	10 (5.8)	12 (6.8)
	20-60	3 (50.0)	122 (70.9)	125 (70.2)
	> 60	1 (16.7)	40 (23.3)	41 (23.0)
Mean age		29.17±19.36	45.91±16.97	45.60±17.26
Residence				
	Salvador and Metropolitan area	3 (50.0)	121 (70.3)	124 (69.7)
	Other urban cities of the state of Bahia	0	37 (21.5)	37 (20.8)
	Rural area	3 (50.0)	14 (8.1)	17 (9.6)
Education level				
	No formal education	0	14 (8.1)	14 (7.9)
	1st to 4th grade	3 (50.0)	32 (18.6)	35 (19.7)
	5th to 8th grade	3 (50.0)	33 (19.2)	36 (20.2)
	Incomplete high school degree	0	28 (16.3)	28 (15.7)
	High School degree	0	51 (29.7)	51 (28.7)
	University level education	0	14 (8.1)	14 (7.9)
Monthly income				
	Up to ½ MW*	3 (50.0)	12 (7.0)	15 (8.4)
	½ MW to <1 MW	1 (16.7)	98 (57.0)	99 (55.6)
	≥ 1 MW up to 2 MW	2 (33.3)	62 (36.0)	64 (36.0)
Sanitation conditions (yes)				
	Piped water	2 (33.3)	147 (85.5)	149 (83.7)
	Residential water filter	2 (33.3)	135 (78.5)	137 (77.0)
	Sewage system and/or septic tank	2 (33.3)	155 (90.1)	157 (88.2)
	Paved streets	2 (33.3)	137 (79.7)	139 (78.1)
	Bathroom inside the residence	2 (33.3)	156 (90.7)	158 (88.8)
	Sink in bathroom	2 (33.7)	146 (84.9)	148 (83.1)
	Garbage collection service	2 (33.7)	143 (83.1)	145 (81.5)
	Habit of walking barefoot	4 (66.7)	25 (15.5)	29 (16.3)

*MW: minimum wage in Brazilian Real, BRL = 954.00 or United State Dollar, USD = 247.00 for December, 2018

Among the HTLV-1 individuals, six were coinfected with *S. stercoralis*. From these, 83.3% (5/6) were males, 50% (3/6) lived in rural areas of the state of Bahia, and 66.7% (4/6) reported walking barefoot regularly (table 1). All HTLV- 1 individuals from the rural area had low socioeconomic conditions and lived in poor sanitary conditions with no access to the sewage system or potable water (data not shown).

### Parasitological diagnosis

The overall frequency of infection by enteroparasites was 23% (41/178) with 15.2% (27/178) of monoparasitism and 7.9% (14/178) of polyparasitism. The most frequent helminths were *A. lumbricoides*, *T. trichiura,* hookworm, and *S. stercoralis* (6.7%, 12/178; 5.1%, 9/178; 3.9%, 7/178, and 3.4%, 6/178, respectively). The pathogenic protozoa *Giardia duodenalis* was found in 2.8% (5/178) of individuals. Other non-pathogenic protozoa were more prevalent, such as *Endolimax nana* (10.1%; 18/178) and *Entamoeba coli* (6.2%; 11/178) (table 2).

**Table 2:** *Strongyloides stercoralis* and other intestinal parasitic infections in HTLV-1 patients (n=178)

**Parasite infection**		Group 1 - urban areas n=161 n (%)	Group 1 - urban areas n=161 n (%)	Total n=178 n (%)
Positive		26 (16.1)	15 (82.2)	41 (23.0)
Monoparasitism		21 (13.0)	6 (35.3)	27 (15.2)
Polyparasitism		5 (3.1)	9 (52.9)	14 (7.9)
Negative		135 (83.9)	2 (11.8)	137 (77.0)
Helminths				
	*Ascaris lumbricoides*	3 (1.9)	9 (52.9)	12 (6.7)
	*Trichuris trichiura*	1 (0.6)	8 (47.1)	9 (5.1)
	Hookworm	3 (1.9)	4 (23.5)	7 (3.9)
	Strongyloides stercoralis*	3 (1.9)	3 (17.6)	6 (3.4)
	*Enterobius vermicularis*	0	5 (29.4)	5 (2.8)
	*Schistosoma mansoni*	1 (0.6)	0	1 (0,6)
	Protozoa			
	*Endolimax nana*	14 (8.7)	4 (23.5)	18 (10.1)
	*Entamoeba coli*	5 (3.1)	6 (35.3)	11 (6.2)
	*Giardia duodenalis*	0	5 (29.4)	5 (2.8)
	*Iodamoeba butschlli*	2 (1.2)	0	2 (1.1)
	*Chilomastix mesnili*	0	1 (5.6)	1 (0.6)
	*Entamoeba histolytica/díspar*	1 (0.6)	0	1 (0.6)

*Three HTLV-1 and S. *stercoralis* coinfected patients belonged to the same family and had *S. stercoralis* hyperinfection (>3.000 larvae/gram of fecal sample). The other three infected individuals had low parasite load (<5 larvae/gram of stool).

*S. stercoralis* was diagnosed in 3.4% (6/178) of the study population. When participants were separated into groups 1 (urban areas) and 2 (rural areas), the infection rate was 1.9% (3/161) and 17.6% (3/17)*,* respectively. The total frequency of other parasites in individuals in the rural areas was 88.2% (15/17) while in individuals from urban areas, the parasitic frequency was 16.1% (26/161). The most frequent pathogenic helminths in individuals from rural areas were *Ascaris lumbricoides* 52.9% (9/17), *Trichuris trichiura* 47.1% (8/17), *Enterobius vermicularis* 29.4% (5/17) while in urban areas *S. stercoralis*, hookworms, and *Ascaris lumbricoides* had a low frequency of 1.9% (3/161 each). Helminth eggs including *E. vermicularis* were diagnosed using the Hoffman and Pons and Janer parasitological methods. The pathogenic protozoa *G. duodenalis* was found in 29.4% (5/17) of individuals from rural areas and none in urban areas (table 2).

Three HTLV-1 individuals with *S. stercoralis* were from the same family and lived in a rural area located on the Southern Coast of Bahia. They presented a parasitic hyperinfection as evidenced by the presence of more than 3,000 larvae/gram of stool quantified by the Baermann-Moraes method with both rhabditiform and filariform larval stages in feces. Additionally, one of these three individuals had free-living males and females, as well as *Strongyloides* eggs released into the stool. The other three infected individuals had low parasite load and discharged <5 larvae/gram of stool (table 2).

### Detection of anti-Strongyloides stercoralis IgG antibodies

**Figure 1. A.**
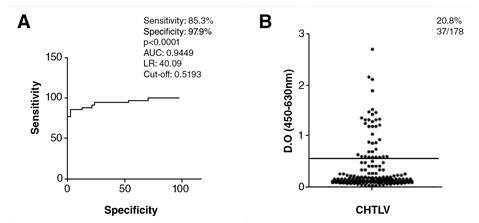
ROC curve indicating the best cut-off point, sensitivity, specificity, area under curve. B.LISA for the detection of serum levels of IgG anti-S. stercoralis in HTLV-1 individuals

To evaluate the exposure of HTLV-1 patients to *S. stercoralis* infection*,* specific IgG antibodies were analyzed in sera. The IgG-ELISA showed 85.29% (42/47) sensitivity and 97.87% (23/24) specificity. *S. stercoralis* IgG antibodies were detected in 20.8% (37/178) of HTLV-1 individuals (figure 1). All six patients with the presence of larvae in their feces were also positive for the *S. stercoralis* IgG-ELISA. All of the 17 subjects who lived in rural areas had specific IgG detected by ELISA.

## Discussion

We found a frequency of 3.4% *S. stercoralis* infection in HTLV-1 individuals from Bahia treated at a specialized medical center. The association between *S. stercoralis* and HTLV-1 was first reported in Okinawa, Japan ^33^. Since then, *S. stercoralis* infection prevalence has been found to be at least 2.4 times higher in individuals infected with the virus than in uninfected individuals ^12^^,^^18^^,^^34^^,^^35^. Moreover, it has been demonstrated that strongyloidiasis increases the risk of the development of HTLV-1-associated diseases, for example, adult T-cell leukemia/lymphoma ^36^. Studies conducted in Brazil have also demonstrated high rates of *S. stercoralis* infection (around 12 to 14%) in association with HTLV-1 ^20^^,^^21^.

Although they came from low-income families, most HTLV-1 subjects lived in the city of Salvador or in other urban areas of cities in the state of Bahia where basic urban amenities are available: treated potable water supply, sewage system connection, regular rubbish disposal, and paved streets and sidewalks. In contrast, the individuals living in rural areas were from poor families with very low incomes.

Besides *S. stercoralis*, other geohelminths such as *A. lumbricoides*, *T. trichiura,* and hookworm were found in the parasitological examination of HTLV-1 patients. These geohelminths were more frequently found in individuals living in rural areas. Several studies have shown that precarious living conditions such as lack of access to basic sanitation, health services, and schooling are the main determinants in the acquisition of intestinal parasitic infections, which continue to represent a major threat to public health in rural areas, as well as in peripheral regions next to urban zones ^37^^,^^38^. In this sense, the epidemiological triangle for the development of parasitic diseases involves the host health status, the parasite, and the environmental conditions ^39^^,^^40^.

The frequency of specific *anti-S. stercoralis* antibodies was 20.8%, which was much higher than the prevalence of larvae in feces. Souza, et al. ^41^ demonstrated a seroprevalence of 16.0% of *S. stercoralis* antibodies contrasting with 1.3% of positive parasitological diagnosis in individuals with lupus erythematosus. Conversely, frequencies of specific antibodies and *S. stercoralis* larvae in feces were very similar in alcoholic individuals, 22.0% and 23.5%, respectively, with a high agreement between the diagnostic methods ^42^. These divergent results could be explained by the continuous exposure to *S. stercoralis* infections by individuals living in endemic areas due to precarious hygiene habits and/or sanitary conditions.

Three individuals with a parasitological diagnosis of *S. stercoralis* were considered hyperinfected with one presenting all parasite evolutionary forms in feces. Factors linked to genetics and host immune response can trigger the infection and determine the severity of strongyloidiasis in individuals with HTLV. HTLV-1 coinfection induces a strong activation of the immune system. The exacerbated production of IFN-γ and TNF-α induced by HTLV-1 infection may negatively modulate the Th2-type cellular response and, consequently, decrease the levels of the main immune mediators involved in the defense against *S. stercoralis* such as IL-4, IL-5, and IL-13, and IgE ^43^^-^^46^. The analysis of serum cytokines in one child with HTLV-1 and *S. stercoralis* hyperinfection showed no alterations except for a significant increase in IL-17 levels following strongyloidiasis treatment ^13^. This could reflect inhibition of HTLV-1 inflammation response by *Strongyloides* in coinfected patients, although a larger number of individuals should be studied to evaluate the immunomodulation in HTLV-1 and *S. stercoralis* coinfection by IL-17.

In conclusion, our study suggests that HTLV-1-infected people living in poverty with precarious sanitary conditions are more predisposed to develop severe forms of *S. stercoralis* infection. Considering the high susceptibility and unfavorable outcome of the infection in these individuals, early diagnosis using parasitological and immunological methods and prompt treatment are critical for the successful management of strongyloidiasis in HTLV-1 carriers, especially those living in rural areas. Additionally, public policies are necessary to improve access to health services and basic sanitation for individuals at high risk of developing severe strongyloidiasis, such as HTLV-1-patients.
